# Ribosome biogenesis rate, a parameter of sensitivity to chemotherapeutic drugs inhibiting rRNA synthesis

**DOI:** 10.3389/or.2025.1740261

**Published:** 2026-01-22

**Authors:** Davide Treré, Lorenzo Montanaro, Massimo Derenzini, Claudio Agostinelli, Enrico Derenzini

**Affiliations:** 1 IRCCS Azienda Ospedaliero-Universitaria di Bologna, Bologna, Italy; 2 Department of Medical and Surgical Sciences (DIMEC), University of Bologna, Bologna, Italy; 3 University of Bologna, Bologna, Italy; 4 Oncohematology Division, European Institute of Oncology IRCCS, Milano, Italy; 5 Department of Health Sciences, University of Milan, Milan, Italy

**Keywords:** cancer, chemotherapy, MDM2, p53, ribosomal proteins, ribosome biogenesis, rRNA synthesis

## Abstract

Many drugs currently used in cancer chemotherapy exert their toxic action mainly by inhibiting ribosome biogenesis (RiBi). This is due to the fact that after inhibition of rRNA transcription ribosomal proteins, no longer used for ribosome building, bind to and neutralize the activity of the murine double minute 2 protein (MDM2, HMD2 in humans), thus hindering cell proliferation and possibly inducing apoptotic cell death. Here, we discuss the existing literature showing how RiBi rate and genomic alterations of ribosomal proteins (RP mutations/deletions) influence the degree of MDM2 inhibition after treatment with RiBi inhibitors in cancer cells. There is evidence that a high RiBi rate is associated with a high RPs release with strong inhibition of MDM2 activity and consequent induction of apoptotic cell death in response to RiBi inhibitors, whereas a low RiBi rate or RP mutations/deletions are associated with a degree of MDM2 inhibition insufficient to kill cancer cells. In the latter case, in cells with wild type p53, association with drugs which stabilize p53 with different mechanisms may overcome cancer cells resistance to RiBi inhibition, whereas in cancers lacking functional p53 addition of MDM2 inhibitors should be considered. From this, the necessity to evaluate the rate of ribosome biogenesis together with the presence of RP mutations/deletions in cancer tissues for predicting the sensitivity of cancer cells to RiBi inhibitors in order to choose more appropriate therapeutic protocols.

## Introduction

The pioneering observation, published in 1946, that nitrogen mustard induced a marked regression of the tumor masses in lymphoma patients, opened a research field on chemical substances which could be used for cancer treatment. From then on, a large number of chemotherapeutic drugs have been tested with the aim to cure neoplastic diseases (1). On the basis of their mechanism of action chemotherapeutic agents are mainly distinguished in DNA alkylating and intercalating agents, antimetabolic agents, topoisomerase inhibitors, kinase inhibitors, mitosis inhibitors, proteasome inhibitors and translation inhibitors (2). However, it is well known that chemotherapeutic treatments not always induce a durable tumor response, drugs resistance being in fact the cause of tumor relapse or progression [reviewed in (3, 4)]. Many factors are at the basis of chemoresistance (4).

- quantitative different drugs uptake by cancer cells;
- presence of drug efflux pumps which hinder the transportation of drugs into the cell;
- genetic changes which render cancer cell less sensitive to chemotherapy (such as for example the mutation of the tumor suppressor *TP53* or overexpression of the anti-apoptotic protein Bcl‐2);
- modifications of the DNA damage repair pathways which reduce drug-induced DNA damage;
- presence of cancer stem cells which are inherently resistant to chemotherapy;
- induction of senescence by chemotherapy that can be incomplete and reversable, with the treatment resistant clones escaping cell cycle arrest and inducing disease relapse.

To these well-established causes of cancer cell chemotherapy resistance, recent data indicate that quantitative and qualitative changes of ribosome biogenesis must be also taken into consideration as factors influencing the chemotherapy efficacy. This derives from the fact that a mayor mechanism, by which many of the drugs used in cancer chemotherapy act, is constituted by the inhibition of ribosome biogenesis, whatever the different primary damage induced by drugs in cancer cells as demonstrated by Burger et al. (5) and recently exhaustively reviewed by Zisi et al. (6). See also Table 1 showing how many of the chemotherapeutic agents currently used in cancer therapy induce a marked inhibition of ribosome biogenesis.

**TABLE 1:** Chemotherapeutic drugs inhibiting ribosome biogenesis.

Drug (generic name)	Drug category	Main clinical use	Main effect on ribosome biogenesis	Mechanism of action on RiBi	Clinical trial ID(s)
CX-5461 (pidnarulex)	Investigational small molecule	Hematologic and solid tumors	Inhibits rDNA transcription (pol I initiation)	Binds GC-rich rDNA/G-quadruplex structures and blocks assembly of the pol I transcription initiation complex, causing selective inhibition of 47S pre-rRNA synthesis and nucleolar stress with p53 activation (6, 7)	Phase I/II studies as pol I/RiBi inhibitor: NCT02719977, NCT04890613, NCT06606990, NCT07069699, NCT07137416
PMR-116	Investigational second-generation pol I inhibitor	​	Inhibits rDNA transcription (pol I promoter escape)	Selective pol I inhibitor that stalls pol I at the rDNA promoter, preventing promoter escape and elongation; potently suppresses rRNA synthesis and tumor growth in MYC-driven models	Ongoing phase Ia/Ib basket trial in advanced solid tumors: ACTRN12620001146987
BMH-21	Preclinical small-molecule pol I inhibitor	​	Inhibits rDNA transcription (pol I elongation)	GC-rich rDNA-binding compound that directly blocks pol I transcription elongation and induces proteasomal degradation of the catalytic subunit RPA194, causing strong RiBi stress (8, 9)	No registered clinical trial as of December 2025 (preclinical only)
CX-3543 (Quarfloxin)	Investigational pol I/G-quadruplex-targeting drug	​	Inhibits rDNA transcription (via nucleolin–G4 disruption)	Fluoroquinolone derivative that binds rDNA G-quadruplexes and disrupts nucleolin–G4 complexes in rDNA, leading to selective inhibition of pol I-driven rRNA synthesis and nucleolar disintegration (10)	Phase I in advanced solid tumors/lymphomas: NCT00955786 Later phase II in carcinoid/neuroendocrine tumors (development subsequently discontinued)
Actinomycin D (dactinomycin)	Classical cytotoxic antibiotic	Sarcomas, pediatric tumors	Strong inhibition of rDNA transcription (pol I elongation)	At low nanomolar doses preferentially binds GC-rich rDNA and blocks pol I transcriptional elongation, rapidly suppressing 47S pre-rRNA synthesis, disrupting nucleoli and stabilizing p53 (“nucleolar stress”) (5, 6)	Long-approved chemotherapeutic; hundreds of trials across indications – no single RiBi-specific NCT usually cited
5-Fluorouracil (5-FU)	Antimetabolite	Solid tumors (GI, breast, others)	Inhibits late steps of pre-rRNA processing	Incorporated into pre-rRNA and small nucleolar RNAs, interfering with site-specific rRNA processing and maturation; classified by Burger et al. (5) as a late rRNA processing inhibitor	Widely approved; many trials in standard regimens (e.g. FOLFOX, FOLFIRI). No single RiBi-focused trial number
Oxaliplatin	Platinum cross-linker	Colorectal and GI cancers	Inhibits rDNA transcription (pol I) and triggers nucleolar stress	Besides forming DNA adducts, specifically suppresses 47S pre-rRNA synthesis and nucleolar integrity at clinically relevant doses; identified as pol I/RiBi inhibitor in cell-based screens (5, 6)	Standard component of FOLFOX, FOLFIRINOX, etc.,; multiple phase III trials, but not designed around RiBi as primary endpoint
Cisplatin	Platinum cross-linker	Many solid tumors	Inhibits rDNA transcription (pol I)	Cross-links rDNA and other genomic DNA, suppressing pol I-mediated transcription and contributing to nucleolar damage and RiBi inhibition (6)	Long-approved cytotoxic; countless trials; no specific RiBi-labelled NCT.
Doxorubicin	Anthracycline	Breast, lymphomas, sarcomas	Inhibits rDNA transcription (pol I) and damages nucleoli	Intercalates DNA and inhibits topoisomerase II; Burger et al. (5) showed inhibition of 47S pre-rRNA synthesis and nucleolar disruption at cytotoxic doses, indicating pol I/RiBi targeting	Standard drug in many regimens (ABVD, CHOP, AC, others); numerous trials, not specifically RiBi-focused
Mitoxantrone	Anthracenedione	Leukemias, breast, prostate	Inhibits rDNA transcription (pol I)	DNA-interacting topoisomerase II inhibitor; like doxorubicin, suppresses 47S pre-rRNA synthesis and nucleolar integrity, classed as pol I transcription inhibitor by Burger et al. (5)	Approved; multiple trials in leukemia, breast and prostate cancer
Methotrexate	Antimetabolite	Leukemias, lymphomas, solid tumors	Functionally inhibits rDNA transcription (via nucleotide depletion)	Depletes purine/pyrimidine pools by blocking dihydrofolate reductase; this limits nucleotide availability for rRNA synthesis and was shown to inhibit 47S pre-rRNA transcription and nucleolar structure (5, 6)	Approved; many standard regimens; no single RiBi-specific trial number
Camptothecin/topotecan	Topoisomerase I inhibitors	Ovarian, lung, leukemias	Inhibits early pre-rRNA processing	In addition to DNA damage, camptothecin family drugs reduce early processing of 47S pre-rRNA (e.g. cleavage at 5′ETS), classified as “early rRNA processing” inhibitors in RiBi assays (5)	Approved agents (e.g. topotecan) with many trials; no dedicated RiBi-trial IDs
Flavopiridol (alvocidib)	CDK inhibitor	Investigational, relapsed leukemias	Inhibits early rRNA processing	CDK9 inhibitor that perturbs transcription of small nucleolar RNAs and rRNA-processing machinery (5,6); Burger et al. (5) classify it as inhibitor of early rRNA processing and show strong synergy with 5-FU on rRNA processing inhibition	Multiple early-phase trials in leukemias; trial numbers depend on indication (e.g. several NCTs in AML/CLL)
Roscovitine (seliciclib)	CDK inhibitor	Investigational	Inhibits early rRNA processing	Inhibits CDK1/2/7/9; alters nucleolar structure and rRNA processing, partly through reduced production of snoRNAs and RiBi factors (5, 6)	Early-phase oncology trials (various NCTs); not specifically developed as RiBi drug
Homoharringtonine/omacetaxine mepesuccinate	Plant alkaloid	Approved for CML (2nd line)	Inhibits late rRNA processing and translation	Classified by Burger et al. (5) as inhibitor of late rRNA processing; also directly blocks translation elongation on ribosomes, leading to depletion of short-lived oncoproteins	Approved for TKI-resistant CML (NCT00462943 and related CML trials); RiBi not explicit endpoint

This table was initially produced by generative AI (ChatGPT EDU, 5.1) and then carefully revised by the authors. List of abbreviations in order to appearance in the Table: NCT, national clinical trial, assigned by ClinicalTrials.gov.; ACTRN, australian clinical trials registration number.; GI, gastrointestinal.; FOLFOX, chemotherapy combination of Folinic acid, Fluorouracil, and OXaliplatin.; FOLFIRI, chemotherapy combination of Folinic acid, Fluorouracil, and Irinotecan.; FOLFIRINOX, chemotherapy combination of Folinic acid, Fluorouracil, Irinotecan and OXaliplatin.; ABVD, chemotherapy combination of Adriamycin, Bleomycin, Vinblastine, and Dacarbazine.; CHOP, chemotherapy combination of Cyclophosphamide, Doxorubicin, Vincristine, and Prednisone/Prednisolone.; AC, chemotherapy combination of Adriamycin and Cyclophosphamide.; AML, acute myeloid leukemia.; CLL, chronic lymphocytic leukemia.; TKI-resistant CML, chronic myeloid leukemia no longer responds to Tyrosine Kinase Inhibitors.

Indeed, targeting ribosome biogenesis was suggested to represent a specific and efficient chemotherapeutic strategy to fight cancer (6, 11–14).

**FIGURE 1:**
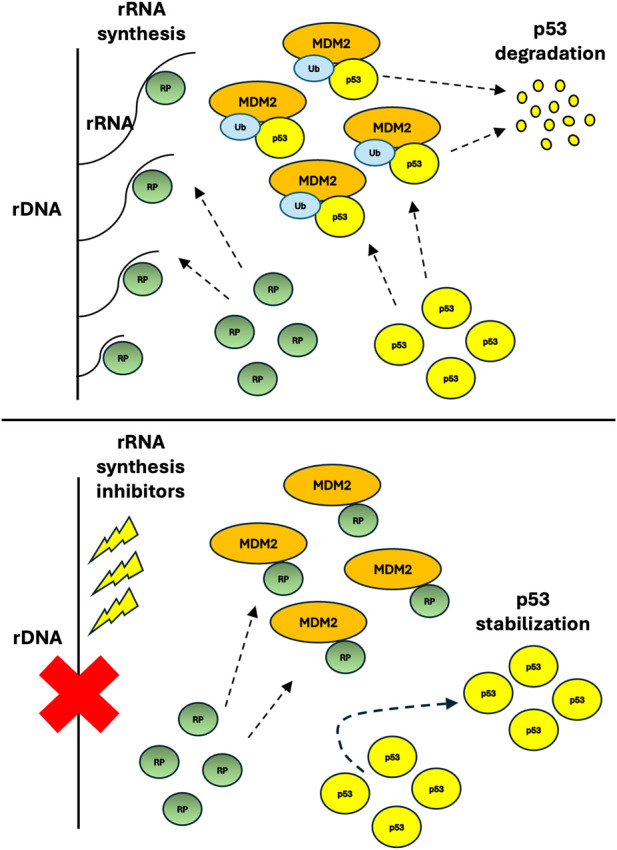
Simplified scheme of the RP–MDM2–p53 pathway. When rDNA transcription and ribosome biogenesis are active (top panel), ribosomal proteins are incorporated into nascent ribosomes and MDM2 can freely ubiquitinate p53, targeting it for degradation. When rDNA transcription is inhibited (bottom panel), for example following chemotherapeutic treatments, ribosomal proteins, which are no longer incorporated into ribosomes, bind to MDM2, thus inhibiting p53 ubiquitination and allowing p53 to accumulate and activate its transcriptional program (RP, ribosomal protein; Ub, ubiquitin).

It is now well established that when rRNA production is downregulated as it occurs after treatment with rRNA synthesis inhibitors, the ribosomal proteins no longer used for ribosome building bind to and neutralize the pro-proliferative activities of the murine double minute 2 (MDM2; and HDM2 in humans) oncogene, mainly by inducing the stabilization of p53 [reviewed in (15, 16), see also Figure 1]. Existing data indicate that the degree of MDM2 inhibition upon treatment with drugs inhibiting rRNA production depends on the amount of ribosomal proteins (no longer used for ribosome building) which bind and inhibit MDM2 activity: greater the amount of available ribosomal proteins greater the level of MDM2 inhibition (17).

Somatic mutation of ribosomal proteins that frequently occurs in cancer cells may be also the cause of a reduced inactivation of MDM2 (18, 19). In the present review we focused our attention on available data reporting how the quantitative and qualitative features of ribosome biogenesis of cancer cells may influence the degree of MDM2 inhibition after treatment with drugs inhibiting ribosome biogenesis, with consequent different levels of response to chemotherapeutic agents, and how these characteristics may be important to establish appropriate chemotherapeutic protocols.

### The efficacy of drugs inhibiting ribosome biogenesis is based on a strict relationship between ribosome biogenesis and cell proliferation

Ribosome biogenesis and cell proliferation are two biological phenomena which are tightly linked each other. Indeed, the molecular mechanism which are activated in cells in order to proliferate also induce a stimulation of ribosome biogenesis whereas induction of ribosome biogenesis downregulation causes arrest of cell cycle and apoptotic cell death. MDM2 activity is an important regulator of the interaction between ribosome biogenesis and cell proliferation.

Ribosome biogenesis is a complex metabolic process [reviewed in (20, 21)] which leads to the formation of the ribosomal particles, constituted by four types of ribosomal RNA (rRNA) and about eighty different ribosomal proteins. These ribonucleoprotein particles, with a diameter of 25–30 nm, are located free or membrane-bound in the cytoplasm where they are engaged in protein synthesis. Transcription of ribosomal DNA occurs in the nucleolus where ribosomal genes are located during interphase. Ribosomal DNA is transcribed by RNA polymerase I (Pol I) to produce the 47S rRNA precursor. The assembly of a specific multiprotein complex at the rDNA promoter containing Pol I is necessary for the initiation of 47S pre-rRNA synthesis. Within this multiprotein complex, at least three basal factors - the ribosomal DNA transcription factor Rrn3 (22) [also referred to as Transcription Initiation Factor I (TIF-I) A (23)], Selectivity factor 1 (SL1), and Upstream Binding Factor (UBF) - are necessary for ribosome gene transcription in mammals (24).

Site-specific modifications of 47S rRNA and processing give rise to the mature 18S, 5.8S and 28s rRNAs. Another kind of rRNA, the 5S rRNA, is produced in the nucleoplasm by the RNA polymerase III and then imported in the nucleolus. Always within the nucleolus, the rRNAs are assembled with the ribosomal proteins (RPs) thus forming the small 40S and the large 60S subunits of the ribosomes: the small 40S sub-unit is constituted by one 18S rRNA and 33 ribosomal proteins (RPS) whereas the large 60S sub-unit is constituted by one each of the 28s, 5.8 and 5S rRNAs together with 47 ribosomal proteins (RPL) (24, 25). There is evidence that the 5S rRNA forms a pre-ribosome complex with RPL5 and RPL11 (26–28). The ribosomal proteins, whose mRNA is transcribed by the RNA polymerase II, are produced in the cytoplasm and then imported in the nucleus (29, 30). The large and the small subunits migrate in the cytoplasm where they give rise to the 80S ribosomal particles. More than 150 non-ribosomal proteins are also involved in ribosome formation together with hundreds of snoRNA (25, 31).

When a cell is stimulated to divide, protein synthesis must greatly increase in order to produce structural and functional components which are necessary to generate normal-sized viable cells (32). This is accomplished by increasing the rate of the biosynthetic mechanisms leading to the formation of ribosomes. The mitogens and the growth factors that stimulate cell proliferation activate the extracellular signal-regulated kinase (MAPK/ERK) pathway that activates (a) Pol I transcription through the phosphorylation of UBF (33, 34) and (b) Pol III transcription, through the phosphorylation of TFIIIB (35). Mitogens and growth factors also activate the PI3K/AKT pathway, which, together with the activated MAPK pathway, enhance Myc-mediated transcription (36). Myc is a major controller of ribosome biogenesis: it stimulates Pol I activity by favoring the recruitment of SL1 to promoters, increases ribosomal protein synthesis by enhancing Pol II transcription, and stimulates Pol III transcription by activating the transcription factor TFIIIB (37–39). Moreover, mitogenic growth factors trigger the pathway of the mammalian target of rapamycin (mTOR), which induces Pol I transcription by activating UBF and TIF-IA, and Pol III transcription by promoting the association of the transcription factors TFIIIB and TFIIIC with 5S rRNA genes (40).

Stimulation of ribosome biogenesis in proliferating cells is also due to the phosphorylation of the tumor suppressor retinoblastoma protein (pRb), which positively controls the transition from G1 to S phase (41). In fact, in its active no phosphorylated form, pRb inhibits rRNA synthesis by binding to UBF (42–46). During cell cycle progression the phosphorylation of pRb hinders the binding to UBF with consequent increase of rRNA transcription rate.

It is now well established that a deranged ribosome biogenesis hinders the normal progression throughout the cell cycle phases. The first demonstration that an altered ribosome biogenesis may block cell proliferation was given by Volarevic et al. (47) who showed that conditional deletion of the gene encoding 40S ribosomal protein S6 in the liver of adult mice abolished the biogenesis of 40S ribosomes and blocked hepatocyte proliferation after partial hepatectomy. The authors concluded that the abrogation of 40S ribosome biogenesis may induce a checkpoint control that prevents cell cycle progression. The nature of the check-point activated by a defective ribosome biogenesis was then clarified by Pestov et al. (48) analyzing the effects of the expression of Bop1D, an amino-terminally truncated Bop1 (block of proliferation) that acts as a dominant negative mutant, in asynchronously growing cells. They found that the expression of Bop1D results in inhibition of 28S and 5.8S rRNA formation, deficiency of newly synthesized 60S ribosomal subunits and cell cycle arrest in G1 phase. Inactivation of functional p53 abrogated this Bop1D-induced cell cycle arrest thus demonstrating the role of p53 in cell cycle arrest consequent to perturbed ribosome biogenesis. They introduced the concept of nucleolar stress, which indicates any perturbation in the nucleolar ribosome biosynthetic machinery that activates a p53-mediated cell cycle checkpoint in proliferating cells. The nucleolar disruption (intended as the redistribution and/or the leakage of the ribonucleoprotein nucleolar components) was then indicated as a common, necessary way for p53 stabilization after nuclear DNA damage and other p53-inducing cell stresses (49). However, as a matter of fact, if perturbed ribosome biogenesis is a necessary step for many cell stresses to cause p53 stabilization, nucleolar disruption is not. Indeed, a downregulation of rRNA synthesis induced by silencing the *POLR1A* gene coding for the RNA polymerase I catalytic subunit stabilized p53 without inducing structural changes in the nucleolar components or leakage of the nucleolar proteins to the nucleoplasm. Worth noting, simultaneous inhibition of rRNA and protein synthesis did not induce p53 stabilization demonstrating that, upon rRNA synthesis inhibition, the p53 stabilization occurred for the unbalance between rRNA and protein synthesis (50).

Stabilized p53 stimulates the transcription of the gene coding for p21Waf1/Cip 1 (51), which inhibits the cyclin-dependent kinases responsible for pRb phosphorylation thus triggering cell cycle arrest at G1 phase (52). Activation of the p53/p21/pRb pathway also induces a G2 arrest sustained through an initial inhibition of cyclin B1-Cdc2, the cyclin-dependent kinase required to enter mitosis, followed by a marked decrease in cyclin B1 and Cdc2 levels (53, 54). Furthermore, stabilized p53 increased the expression of PUMA and BAX which, by antagonizing the anti-apoptotic activity of Bcl-2 protein, induce programmed cell death (55–58). Interestingly, stabilized p53 also controls ribosome biogenesis: in fact, p53 inhibits both Pol I transcription by binding to the selectivity factor SL1 (59, 60), and Pol III transcription by binding to TFIIIB (60), thus inducing a downregulation ribosome biogenesis. Moreover, stabilized p53 inhibits pRb phosphorylation and the non-phosphorylated pRb hinders both rRNA synthesis by binding to UBF (42–46), and Pol III transcription by binding to TFIIIB (43).

**FIGURE 2:**
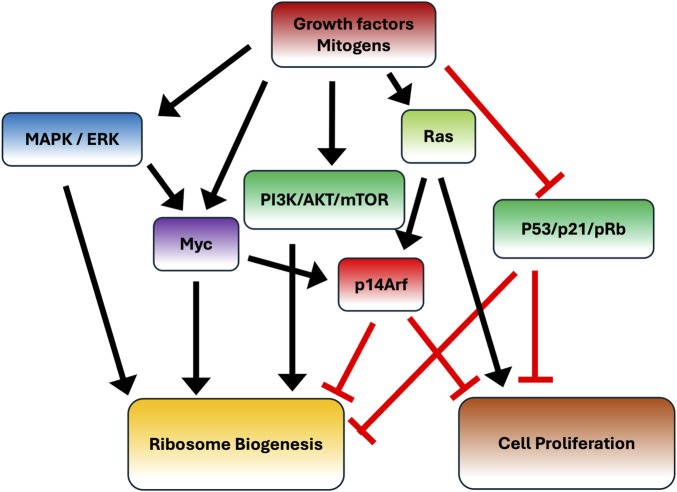
Ribosome biogenesis and cell proliferation. The diagram illustrates the major pathways involved in the control of ribosome biogenesis and cell proliferation, and their interconnections. Black arrows represent activating effects, whereas blunted red lines indicate inhibitory pathways.

The strict relationship between ribosome biogenesis and cell proliferation was also demonstrated by the fact that the tumor suppressor protein p14Arf, which is induced by a series of stress signals, such as hyperproliferative signals emanating from oncogenic Ras and overexpressed Myc (61), other than stabilizing p53 by binding to Mdm2 (62) also negatively regulates rRNA transcription by inhibiting UBF phosphorylation (63). The interplay of the various factors controlling ribosomal biogenesis and cell proliferation is schematically reported in Figure 2.

### Relationship between ribosome biogenesis and cell proliferation: the role of MDM2

MDM2 is an E3 ubiquitin-protein ligase whose major and most investigated function is to ubiquitinate p53 thus marking it for proteasomal degradation (64–66). MDM2 contains four distinct domains which are all necessary for the activity of the E3 ligase: an N-terminal p53-binding domain, a central acidic and a zinc finger domain, and a C-terminal RING (Really Interesting New Gene) domain (67–70). The NH2 terminal domain of MDM2 interacts with an alpha-helix present in the NH2 terminal transactivation domain of p53 (71). More precisely, this interaction has been localized to a small (aa 25–109) hydrophobic pocket domain at the NH2 terminus of MDM2 and a 15-aa amphipathic peptide at the NH2 terminus of p53 (71, 72). It is worth noting that the binding of MDM2 to the NH2 terminal transactivation domain of p53 blocks its transcriptional activity directly (72, 73) which is independent of the MDM2 function as the E3 ligase that ubiquitinates p53 for proteasome degradation (70). Importantly, inhibitors that bind the MDM2 N-terminal domain do not disturb the ubiquitination activity of the MDM2 RING domain present in the C-terminus (74).

The central acidic domain (AD), has a regulatory function; many small proteins bind to the AD and inhibit the MDM2 ubiquitin ligase activity thus inducing p53 stabilization. In this context an important role is played by the tumor suppressor protein p14ARF and by the ribosomal proteins which in fact inhibit the MDM2 ubiquitin ligase activity by binding to its central AD (75–79).

The mechanism at the basis of this inhibitory activity is not yet clarified. It has been suggested that the central acidic domain of MDM2 may act as a flexible arm to juxtapose the N-terminal-bound p53 within close proximity of the C-terminal RING domain in order to facilitate ubiquitin transfer. The binding of the RPs or other protein to the acidic domain may render MDM2 more rigid, thus hindering MDM2 to bring its RING domain and p53 together (15).

Finally, the RING domain is responsible for the hetero-oligomerization of MDM2 with its homologous partner MDMX as well as for homo-oligomerization with other MDM2 molecules. The RING domain also contains the enzymatic activity of MDM2 and catalyzes the ubiquitination of p53. MDM2 harbors a self- and p53-specific E3 ubiquitin ligase activity within its evolutionarily conserved COOH terminal RING finger domain (Zinc-binding), and its RING finger is critical for its E3 ligase activity (79).

Other than p53, MDM2 also controls the activity of a second important tumor suppressor protein, the retinoblastoma-associated protein pRb. There is evidence that the MDM2-pRb interaction results in inhibition of the pRb negative regulatory function on cell growth (81) and that the central acidic domain of MDM2 is essential for pRb interaction (82). Data also indicate that the binding to MDM2 promotes either ubiquitin-dependent degradation (83) or proteasome-dependent ubiquitin-independent degradation of pRb (84).

E2F-1 is another factor whose activity is controlled by MDM2. E2F-1 belongs to a family of transcription regulators (E2Fs) which control the expression of genes whose products are necessary for the entry and passage throughout the S phase (85, 86). E2f1 binds to the central acidic domain of MDM2 (87) and this binding prolongs the half-life of the E2F1 protein by inhibiting its proteasomal-dependent degradation (88).

c-Myc activity too has been shown to be regulated by MDM2. Indeed, MDM2 increases c-Myc mRNA stability and translation whereas MDM2 inhibition renders c-Myc mRNA unstable, and reduces c-Myc protein expression (89).

There is evidence that ribosomal proteins control the activity of MDM2 [reviewed in (90)]. The effect of the binding of RPs to the central acidic domain of MDM2 has been widely investigated mainly considering the degree of p53 stabilization upon ribosome biogenesis stress. The pioneering works by Lohrum et al. (91), Zhang et al. (92), Dai and Lu (93), and Bhat et al. (94) demonstrated that the p53 stabilization after the inhibition of rRNA synthesis was due to the fact that the ribosomal proteins RPL11 and RPL5, no longer used for ribosome building, bind to the central acidic domain of MDM2 thus hindering the MDM2-mediated p53 ubiquitination and degradation. Further studies demonstrated that RPL11 and RPL5 form, together with the 5S rRNA, a stable 5S RNP complex that actually binds to MDM2 thus preventing p53 degradation, all the components of the complex being necessary for its inhibitory function (27, 28, 95). In addition to RPL11 and RPL5, many other ribosomal proteins have been shown to interact with MDM2 with the consequent p53 stabilization, including RPS3 (96), RPS7 (97), RPS14 (98), RPS15 (99), RPS20 (99), RPS25 (100), RPS26 (101), RPS27 (102), RPL22 (103), RPL23 (104), RPL26 (105), and RPL37 (99) [see review by Kang et al. (90)].

In this context it should be considered that the inhibition of rRNA synthesis results in proteasomal degradation of the newly synthesized RPs, but does not affect the ribosome-free L5 and L11 [reviewed in (106)].

The importance of some of these ribosomal proteins in the inhibition of MDM2 upon ribosomal biogenesis inhibition is still to be clarified.

In addition to the main RPs-MDM2-p53 pathway some studies suggest that there is a direct link between RPs and p53 that is independent of MDM2 binding. Indeed, it has been shown that in response to DNA damage, L26 binds to the p53 mRNA 5′-UTR and increases the rate of p53 protein translation [reviewed in (106)].

Other than p53 stabilization, it has been demonstrated that selective inhibition of rRNA synthesis causes a reduction of E2F-1 protein amount in p53-deficient cancer cells with the consequent inhibition of cell cycle progression. E2F-1 downregulation was shown to be due to the release of RPL11 which by binding to MDM2 inactivates its E2F-1 stabilizing function (88) thus allowing E2F-1 proteasomal digestion (107, 108).

Also, the activity of pRb is influenced by RPs released after inhibition of ribosomal biogenesis. Considering that the central acidic region of MDM2 is involved in the binding to pRb (82) with its consequent degradation, the RPs left free to bind to the central acidic region may, in fact, strongly reduce the MDM2-mediated pRb degradation.

More articulated are the relationships between the inhibition of ribosome biogenesis and the c-Myc oncogene expression. c-Myc activity other than being positively controlled by MDM2 (89) is, in fact, regulated by the ribosomal protein L11 which represses c-Myc expression (109). Indeed, overexpression of L11 inhibits c-Myc-induced transcription and cell proliferation, while reduction of endogenous L11 increases these c-Myc activities (109, 110). RPL5 co-operates with RPL11, in mediating the degradation of the c-Myc mRNA, with consequent inhibition of c-Myc activity (111). There is evidence that treatment of cells with the ribosomal stress-inducing agents actinomycin D or 5-fluorouracil significantly decreased c-Myc mRNA levels in an L11-dependent manner (112).

**FIGURE 3:**
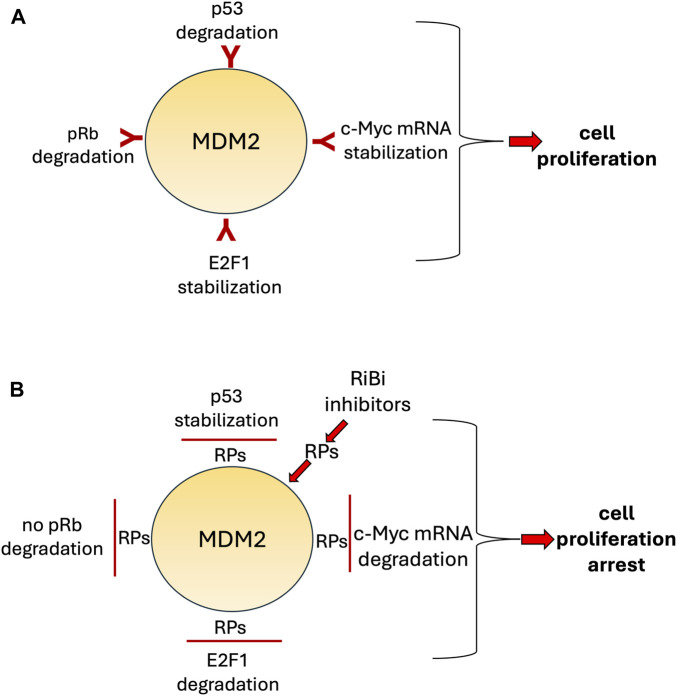
Simplified, schematic representation of the pro-proliferative activities of MDM2 **(A)** and of their neutralization by ribosomal proteins (RPs) released after exposure to inhibitors of ribosome biogenesis (RiBi) **(B)**. MDM2 binds to p53 and pRb and induces their degradation, whereas the binding to E2F-1 and c-Myc mRNA induces their stabilization. These activities were inhibited by RPs which impede the binding of MDM2 to these factors thus causing p53 and pRb stabilization and E2F-1 and c-Myc mRNA degradation.

All these data demonstrate that RPs released after a perturbed ribosome biogenesis induce a neutralization of the pro-proliferative activity of MDM2 by stabilizing p53, by reducing pRb digestion and E2F-1 stabilization and by decreasing c-Myc protein expression (see Figure 3) thus blocking in this way the cell cycle progression and possibly inducing apoptotic cell death.

Is worth noting that the induction of apoptotic cell death depends on the amount of stabilized p53: lower levels of p53 resulting only in cell cycle arrest whereas higher levels resulting also in apoptotic cell death (17, 113).

### The level of p53 stabilization induced by rRNA synthesis-inhibiting drugs depends on the cell ribosome biogenesis rate

The strict relationship between the level of cell ribosome biogenesis and the degree of p53 stabilization upon treatment with drugs inhibiting rRNA synthesis has been demonstrated in a study conducted in a series of human *TP53* wild-type cancer cell lines, treated with a dose of actinomycin D (Act-D) which selectively inhibits rRNA transcription (17). The cell lines used were characterized by different levels of rDNA transcription. Treatment with Act-D reduced the synthesis of rRNA to similar values in all cell lines. However, the level of stabilized p53 was directly related to the baseline level of rRNA transcription (which characterized the cell lines before drug treatment). The same results were obtained using drugs which inhibit ribosomal biogenesis in a different way than Act-D, such as doxorubicin, 5-fluorouracyl (5-FU) and CX-5461. Indeed, Act-D at low concentration intercalates into the GC-rich regions of rDNA thus inhibiting Pol I-mediated transcription (114, 115), doxorubicin is a DNA-intercalating agent which also inhibits rDNA transcription (5), 5-FU is an antimetabolite which disrupts the action of thymidylate synthase (116) and also inhibits ribosomal biogenesis by blocking rRNA processing (5), CX-5461 inhibits ribosome biogenesis by reducing the binding affinity of the SL1 pre-initiation complex and RNA polymerase I complex to rDNA promoters (117–119) and by inhibiting topoisomerase II activity (120), which is involved in the activation of RNA polymerase I transcription by facilitating pre-initiation complex formation activation (121). The reason for the different level of p53 stabilization upon inhibition of rRNA synthesis according to the rate of ribosome biogenesis of the cells was shown to lie in the different amounts of RPs which, no longer used for ribosome building, inhibited MDM2 activity, thus hindering p53 degradation. Immunoprecipitation analysis, in fact, demonstrated that the amount of RPL11 (one of the RPs most involved in MDM2 sequestering) (92, 94), bound to MDM2 after inhibition of rRNA transcription was much higher in cells with a high rate of ribosome biogenesis, as compared to cells with a low ribosome biogenesis activity, with the consequence of a higher p53 stabilization. Furthermore, silencing of *RPL11* expression was found to eliminate the difference in p53 stabilization between cells with high and low rRNA synthesis after inhibition of ribosome biogenesis. Regarding the effect of inhibition of rDNA transcription on cell cycle progression and apoptotic cell death in cells with different rate of ribosome biogenesis, this study provided evidence that inhibition of ribosome biogenesis causes cell cycle arrest in all cell lines independent of the rate of rDNA transcription. This was not the case for apoptotic cell death, occurring only in cells with a high level of ribosome biogenesis, which was due to the induced higher amount of stabilized p53. These results were consistent with those previously reported regarding the relationship between the level of p53 stabilization and apoptosis induction (113).

The strict relationship between the rate of cell ribosome biogenesis and the level of stabilized p53 upon treatment with drugs inhibiting rDNA transcription was absent if p53 stabilization was induced by drugs acting in different ways from the inhibition of ribosome biogenesis, such as hydroxyurea (HU) and MDM2-p53 interaction inhibitors. HU, mostly used in the treatment of chronic myeloproliferative disorders, limits the cellular supply of deoxyribonucleotides by acting as an inhibitor of ribonucleotide reductase (122, 123). Stabilization of p53 by HU was due to the fact that the drug induces phosphorylation of p53 at serine 15 (124) thus preventing the binding of p53 to MDM2 with the consequent stabilization of the tumor suppressor (125).

Regarding inhibitors of the MDM2-p53 interaction, Nutlins were the first small molecules proposed, Nutlin 3 being one of the most efficient among them. Indeed, Nutlin-3 binds MDM2 in the p53-binding pocket with a high affinity thus competing with MDM2 for p53 binding, with consequent p53 stabilization and activation (126, 127). The discovery of Nutlins strongly stimulated the identification of numerous small molecules as inhibitors of the MDM2-p53 interactions with possible clinical application [reviewed in (128–130)].

Scala et al. (17) demonstrated that treatment with either HU or Nutlin-3 induces the same level of p53 stabilization independently of the ribosome biogenesis rate of the cells. Interestingly, exposure of cells with low biogenesis rate to Act D plus HU increased the level of p53 stabilization in comparison with single agent treatment, whereas no increase of p53 stabilization occurred in cells with a high rate of ribosome biogenesis in comparison to Act D treatment alone. Moreover, double drug treatment of cells with low rDNA transcription was found to induce apoptosis which was absent after single drug exposure.

The clinical relevance of ribosome biogenesis rate for the efficacy of drugs inhibiting rRNA transcription has been recently demonstrated in a study carried out in patients with Diffuse large B-cell lymphoma (DLBCL) (131). DLBCL is a hematological malignancy currently treated in first-line with a chemoimmunotherapy protocol including cyclophosphamide, doxorubicin, vincristine and prednisone (CHOP) with the addition of the anti CD20 antibody rituximab. A significant fraction of DLBCL patients (30%–40%) are refractory or relapse after first-line chemoimmunotherapy. Since the majority of DLBCL cases carry a wild-type *TP53*, additional factors may contribute to chemoresistance (132). Indeed, hyperexpression of the anti-apoptotic factor BCL-2 represents one of the foremost relevant chemoresistance mechanisms, being associated with poor prognosis in DLBCL (133, 134).

On the other hand, despite this established role of BCL-2, specific inhibition of its antiapoptotic activity by the BH3-mimetic Venetoclax showed low efficacy in the relapsed/refractory setting of DLBCLs (135, 136), thus indicating that other factors may contribute to the chemoresistance of BCL-2 hyper expressing DLBCLs. Since one of the most important mechanisms of action of doxorubicin and of the metabolic active form of cyclophosphamide, acrolein, consists in the inhibition of ribosome biogenesis (RiBi) with the consequent p53 stabilization (5,137), the authors evaluated the possibility that the reduced sensitivity to chemotherapy of DLBCLs overexpressing BCL-2 could be due to a lower baseline ribosome biogenesis rate compared to DLBCLs characterized by low BCL-2 expression, with the consequence of a lower level of p53 stabilization. Actually, enforced BCL-2 expression was found to reduce the rRNA synthesis both in cells with wild-type or mutated *TP53*. This inhibitory effect was very likely due to a nucleolar stress induced by BCL-2, as demonstrated by the observation of nucleolin delocalization in the nucleoplasm following enforced BCL-2 expression (138–141).

Further experiments carried out using *TP53* wild type DLBCL cells overexpressing BCL-2, demonstrated that the treatment with CHOP, modelled *in vitro* by combining doxorubicin, acrolein (a metabolite of cyclophosphamide known to inhibit rRNA synthesis (137), vincristine and metylprednisone or treatment with single agents RiBi inhibitors doxorubicin, Act-D and CX-5461 induced a level of p53 stabilization that was lower in comparison to that induced in control cells not over expressing BCL-2, with a consequent significant reduction of drug-induced cytotoxic effects. Contemporary treatment with the pro-apoptotic, BH3-mimetic, Venetoclax was unable to completely abrogate BCL-2-mediated pro-survival activity. High level of p53 stabilization and apoptotic cell death were completely repristinated by adding to ribosome biogenesis inhibitors and Venetoclax either Nutlin 3A or etoposide, two substances which stabilize p53 independently of the RP-MDM2-p53 pathway (126, 127, 142). Noteworthy, the effect of these latter drugs on the degree of p53 stabilization was additive and not substitutive of the activity of ribosome biogenesis inhibitors.

The efficacy of the triple combination of ribosome biogenesis inhibitors, with Venetoclax and MDM2 inhibitors was also demonstrated *in vivo* using a *TP53* wild-type, BCL-2-positive DLBCL patient-derived xenograft model. After engraftment, mice were treated with Act-D, Venetoclax and Idasanutlin [a MDM2 inhibitor (143)] as monotherapy or combined in different regimens (Act-D + Venetoclax, Idasanutlin + Venetoclax, Act-D + Idasanutlin, Act-D + Idasanutlin + Venetoclax). The triple combination of Act-D, Venetoclax and idasanutlin exerted synergistic effects, significantly reducing tumor growth as compared to doublets (Act-D + Venetoclax, Idasanutlin + Venetoclax, Act-D + Idasanutlin).

**FIGURE 4:**
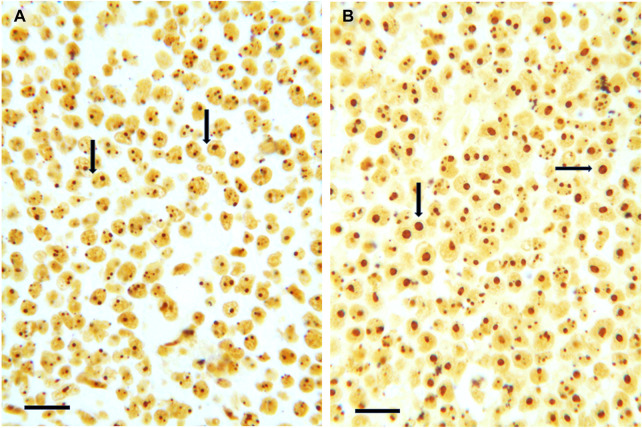
**(A,B)** Histological sections from two routinely processed diffused large B cell lymphoma samples specifically silver-stained for the argyrophilic nucleolar organizer region proteins. The nucleoli are very darkly stained by silver. Note the very small size of nucleoli (arrows) in **(A)** in comparison with the large size of nucleoli (arrows) in **(B)**. Bar, 25 μm.

The relationship between Bcl-2 expression and ribosome biogenesis rate was also demonstrated, in histological sections, from two DLBCL patient cohorts (131) after a silver staining procedure specific for the argyrophilic proteins of the nucleolar organizer regions (AgNOR staining), which are the nucleolar structures where the rRNA synthesis occurs (144–146), and whose distribution is directly related to the nucleolar size and to the rate of ribosome biogenesis (147–149). The area occupied by the silver-stained nucleoli was measured by image analysis: the authors found that the nucleolar size values, and then the ribosome biogenesis rate, were highly variable (Figures 4A,B) and were inversely related to BCL-2 expression. Interestingly, low nucleolar values independently predict a poor outcome following first-line chemoimmunotherapy.

All these data demonstrated that in *TP53* wild-type cells the baseline rate of ribosome biogenesis strongly influences the efficacy of chemotherapeutic drugs acting by inhibiting ribosome biogenesis. Moreover, they indicate that in order to obtain an adequate p53 stabilization with consequent induction of cytotoxic effects in cancer cells with a low ribosome biogenesis rate, it is necessary to combine RiBi inhibitors treatment with drugs stabilizing p53 through different mechanisms (thus by-passing the MDM2-RPs axis).

If the rate of ribosomal biogenesis influences the sensitivity of cancer cells with wild-type TP53, there is evidence that also the response of cancer cells with mutated *TP53* to inhibitors of rRNA synthesis may be influenced by their baseline rRNA transcription level as well. By evaluating the *in vitro* effects of CX-5461 on a panel of 32 established human ovarian cancer cell lines. Sanij et al. (150) shown that there was no statistically significant correlation between *TP53* mutation status and sensitivity to CX-5461 The efficacy of growth inhibition by CX-5461 correlated with a higher rate of basal rDNA transcription in the sensitive compared with the resistant cancer cell lines.

The rate of rRNA transcription may influence the response of chemotherapy in cancers with p53 disruption by modulating the activity of MDM2 on additional factors controlling cell proliferation. It has been in fact demonstrated that selective inhibition of rRNA synthesis causes a reduction of E2F-1 protein amount in p53-deficient cancer cells with consequent inhibition of cell cycle progression. The E2F-1 downregulation was shown to be due to the release of RPL11 which by binding to MDM2 inactivates its E2F-1 stabilizing function (88) thus allowing E2F-1 proteasomal digestion (107, 108).

The rate of ribosome biogenesis may also influence the effects of chemotherapeutic agents inhibiting rRNA synthesis by modulating pRb activity. Considering that the central acidic region of MDM2 is involved in the binding to pRb (82) with its consequent degradation, the amount of RPs left free to bind to the central acidic region may, in fact, strongly inhibits the MDM2-mediated pRb degradation.

The amount of RPs left free after ribosomal biogenesis inhibition, may also directly influence the c-Myc activity. Indeed, the RPs L5 and L11 have been found to be responsible for c-Myc mRNA degradation with the consequent reduction of c-Myc protein synthesis (111) and these effects are enhanced after ribosomal stress induced by RiBi inhibitors (112).

### Qualitative changes of ribosome proteins may hinder the RP-mediated MDM2 inhibition

The heterogeneous composition of ribosomes is a characteristic of cancer cells and mutation of ribosomal proteins has been proposed to induce a preferential translation of some mRNAs which facilitates cancer onset and progression (151, 152). To describe these altered ribosomes which preferentially translate oncogenic and pro-survival genes, the term of onco-ribosomes was introduced (153, 154). The role played by ribosomes carrying mutated ribosomal proteins in tumor development and progression has been reported in exhaustive reviews to which the reader should be addressed (13, 90, 155–157). On the other hand, mutations/deletions of ribosomal proteins may also influence the efficacy of chemotherapeutic treatment with drugs whose function is mainly based on the inhibition of rRNA synthesis.

Somatic mutations of numerous ribosomal proteins have been described in various types of cancer [see (90, 156)] and genome-wide sequencing indicates that gene mutations interesting *RPL5, RPL11, RPL10, RPL22* and *RPS15* are relatively frequent in some cancer types (90, 155, 158).

Heterozygous *RPL5* and *RPL11* mutations or deletions have been described in numerous human cancers. The majority of the *RPL5* and *RPL11* cancer-associated mutations, are missense mutations (66% and 73% in *RPL5* and *RPL11* mutations, respectively) (19). Particularly frequent are the *RPL5* mutations/deletions. In a study carried out in 4,926 samples from 16 cancer types *RPL5* mutations/deletions have been reported in 34% of breast cancer, in 28% of melanoma, in 30% of multiple myeloma and in 2% of T-acute lymphoblastic leukemia (T-ALL) (158). Somatic mutations in *RPL10* are found in ∼8% of pediatric T-ALL (159, 160) and, with a lower frequency (2%), also in multiple myeloma (161).

Inactivation of RPL22 due to heterozygous deletion has been observed in 10% of T-ALL patient samples (162) and *RPL22* mutations have been described in about 10% of gastric, endometrial, and colorectal and adrenocortical solid cancer samples (162–166).

A whole-exome sequencing of samples from patient with chronic lymphocytic leukemia showed that a large proportion of cases (19.5%) harbored mutations in RPS15. All RPS15 mutations represented somatic missense variants. Finally, recurrent somatic mutations and deletions of RP genes have been described in DLBCL (167).

How can the above reported mutations of RPs negatively influence the efficacy of chemotherapeutic drugs inhibiting ribosome biogenesis?

Orsolic et al. (19) evaluated the impact of representative clustered cancer-associated missense *RPL5* mutations on MDM2-mediated degradation of wt p53. They found that RPL5 proteins harboring distinct mutations in the RPL5’s interaction interface with 5SrRNA were unable to cooperate with wt RPL11 in the inhibition of MDM2-mediated p53 degradation. This was particularly evident in cells treated with 5 nM actinomycin D, a drug concentration that selectively inhibits RNA polymerase I, in which the expression of the 5S rRNA inter-acting class of clusters mutants strongly impaired p53 stabilization. Regarding *RPL11* mutations, always Orsolic et al. (19) provided evidence for only a modest role of *RPL11* mutations in the reduction of RP-MDM2-mediated p53 stabilization.

Regarding RPL22 it has been reported (103) that by binding to the central acidic domain of MDM2, this protein can suppress MDM2-mediated p53 ubiquitination and degradation, leading to p53 stabilization and consequent activation. Knockdown of *RPL22* impaired p53 activation by ribosomal stress, indicating its requirement for ribosomal stress activation of p53. Therefore, *RPL22* mutations may reduce the level of p53 stabilization in cancer cells upon treatment with ribosome biogenesis inhibitors.

Regarding RPL10, to our knowledge no data are available on a possible role of *RPL10* mutations on the stabilization of p53 throughout the RP-MDM2-p53 pathway. On the other hand, *RPL10* mutations may lead to a deficit of others RPs of the large ribosome sub-unit which are involved in the control of the RP-MDM2-p53 pathway. Indeed, it has been shown that the depletion of several individual r-proteins in one of the two ribosome subunits caused a decrease in all r-proteins of the same subunit (168).

RPS15 has been demonstrated to bind Mdm2 and activate p53 and the mechanism of Mdm2 and p53 stabilization appears to be through the inhibition of the E3 ubiquitin ligase activity of Mdm2 (95). Indeed, *RPS15* mutants compared with wild-type (wt) *RPS15* showed reduced stabilization and increased p53 degradation (167).

Taken together, all these data strongly suggest that cancers carrying mutations/deletions of the above reported ribosomal proteins may be less sensible to chemotherapeutic agents, which mainly act by hindering the synthesis of rRNA, because of the reduction of the induced RP-mediated MDM2 inactivation. It is worth noting that individual depletion of several r-proteins of the small or large subunit causes a decrease in all r-proteins of the same subunit (168).

### Quantitative and qualitative evaluation of RiBi rate in cancer tissues

**FIGURE 5:**
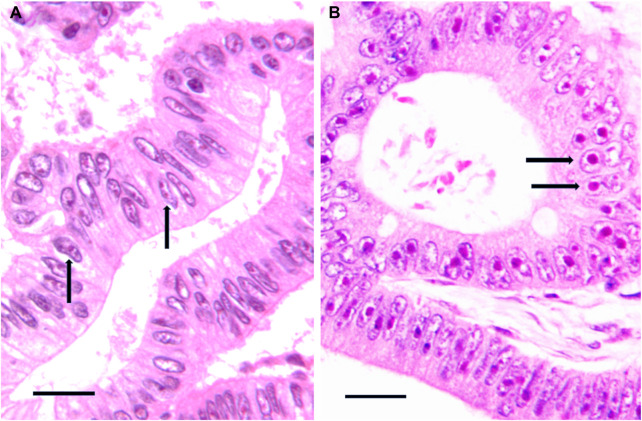
**(A,B)** Histological sections from two routinely processed colon adenocarcinoma samples, stained with hematoxylin and eosin. Nucleoli are mainly stained with eosin due to their high protein content. Note the very small size of nucleoli in **(A)** (arrows) in comparison with the very large, “prominent”, nucleoli in **(B)** (arrows). Bar, 25 μm.

**FIGURE 6:**
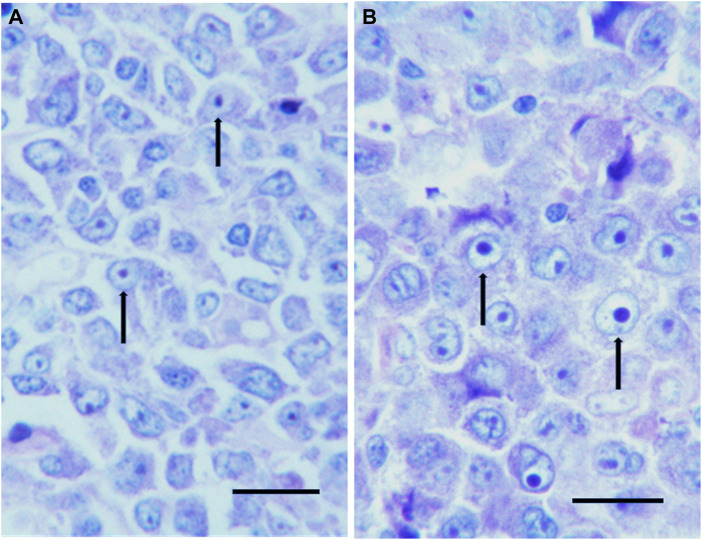
**(A,B)** Histological sections from two routinely processed diffused large B cell lymphoma samples stained with Giemsa. Arrows indicate small nucleoli in **(A)** in comparison with large nucleoli in **(B)**. Bar, 25 μm.

Regarding the rate of ribosome biogenesis there is evidence that this parameter can be easily evaluated in histological sections from routinely processed tissue samples by measuring the nucleolar size and number, (which are strictly related to the rRNA transcription rate), after silver staining of the nucleolar organizer region proteins (Figures 4A,B) [reviewed in (13, 169)]. On the other hand, information about the ribosome biogenesis rate of cancer tissues can be also roughly, but very simply obtained considering the presence or the absence, in histological sections from routinely processed tissue samples stained with hematoxylin and eosin (H&E), of what the pathologists call “prominent nucleoli”. Indeed, in these preparations the nucleoli, due to their high protein concentration, are intensely stained with eosin and then clearly detected. The same is true for hematological cyto-histological preparations stained by Giemsa which is a soluble complex mixture of methylene chloride blue, eosinate methylene blue and eosinate azure II. Giemsa treatment allows nucleoli to be deeply blue stained and therefore to be clearly detected. Interestingly, the size of Giemsa-stained nucleoli is used to drive diagnostic algorithm: large, prominent nucleoli are typical of many aggressive variants of B lymphomas and have a key role in the recognition of Reed-Sternberg cells in classical Hodgkin lymphoma (170). In Figures 5A,B, the high variability of nucleolar size is shown in two different colon cancer samples stained with (H&E), and in two different lymphoma samples stained with Giemsa in Figures 6A,B. Note the prominent nucleoli, morphological expression of a high ribosome biogenesis rate, of cancer samples shown in Figure 5B and in Figure 6B, in comparison with the very small nucleoli of cancer samples shown in Figure 5A and in Figure 6A, indicative of a low ribosome biogenesis rate.

Another procedure, always applied to formalin fixed and paraffin embedded human tissues, has been proposed by Guner et al. (171) which utilizes a chromogenic *in situ* hybridization assay for detecting the expression of the 5′external transcribed spacer of the 45S ribosomal RNA precursor thus evaluating the rRNA polymerase I activity. Also, the proportion of active to inactive rDNA repeats has been suggested as a useful biomarker for sensitivity to targeted Pol I transcription therapies (172). However, as stated by the same authors, a potential barrier to the effectiveness of rDNA chromatin status as a biomarker is the lack of precision with which the proportion of active rDNA repeats can currently be determined.

Recently, an *in silico* approach to systematically assess on transcriptomic data the activity of ribosome synthesis has been proposed. This signature evaluates the expression of over 200 genes implicated in the ribosome production process, which are essential for tumor growth and survival (173). Interestingly, using the expression data from CCLE (Cancer Cell Line Encyclopedia) and the drug sensitivity data from Genomics of Drug Sensitivity in Cancer (GDSC) database, the authors (173) performed an association analysis between ribosome biogenesis activity and the half-maximal response of each drug. They found that cancer cells with higher RiBi activity were more sensitive to 65 drugs. They also performed a differential drug response analysis on the activity of five sub steps of RiBi. Interestingly, they found that cancer cells with heightened activity in any of the sub-steps of RiBi were more sensitive to numerous drugs and 23 drugs exhibited significant differences in drug sensitivity in all differential analyses. Notably, among these drugs were the inhibitors of RiBi methotrexate, 5-fluorouracil and CX-5461.

Finally, quantitative evaluation of fibrillarin mRNA expression was proposed as a surrogate marker of ribosome biogenesis (174) considering the essential role of this protein in ribosome biogenesis (175, 176). Of note, the authors observed that patients carrying tumors with low FBL level displayed poor outcome compared to current clinical gold standards.

In our opinion, at the present time, the more simple and precise procedure to have information on the ribosome biogenesis rate of cancer tissues remains the morphometric analysis of the silver-stained nucleoli [reviewed in (13, 169)], which could be useful to predict the sensitivity of cancer cells to ribosome biogenesis inhibitors.

Anyway, methods which allow the evaluation of the ribosome biogenesis rate in histological sections of cancer tissues should be preferred considering that in these conditions cancer cells can be distinguished from stromal and inflammatory cells whose amount may be highly variable thus variably influencing the RiBi rate value.

Regarding the qualitative changes of the RPs, in order to exhaustively predict the chemosensitivity DNA sequencing for RP mutations/deletions should be also carried out considering that *RPL5, RPL11, RPL10, RPL22* and *RPS15* mutations/deletions frequently occur in many types of cancers (90, 155, 158), and that these proteins are involved in the negative control of MDM2 activity. In this context particular attention should be paid to *RPL5* mutations which have been demonstrated to strongly hinder p53 stabilization after inhibition of ribosome biogenesis (19).

## Conclusion

There is now evidence that the ribosome biogenesis rate and ribosome protein mutations/deletions may greatly influence the sensitivity of cancer cells to drugs inhibiting the synthesis of rRNA and, consequently, the efficacy of their use for chemotherapeutic treatment. This can be considered true both for currently employed RiBi inhibiting - chemotherapeutic drugs and for those that may be developed in the future [see also Supplementary Table S5 in Ogawa et al. (177)]. How the ribosome biogenesis rate and ribosome protein mutations/deletions may condition the response to ribosome biogenesis inhibitors is schematically summarized in Table 2. Inhibition of ribosome biogenesis in cells without RP qualitative changes but with a high ribosome biogenesis rate induces apoptotic cell death independently of the *TP53* status (150, 177). This is not the case if cancer cells are characterized by a low baseline ribosome biogenesis rate or by ribosome protein qualitative changes. We propose that in this set of cancers, the utility of two different chemotherapeutic approaches should be considered: in the case of cancers with wild-type *TP53* other drugs which stabilize p53 independently of the RP-MDM2-p53 pathway (such as, e.g., HU and Etoposide) may be added to RiBi inhibitors in order to induce a p53 stabilization level sufficient for the induction of apoptotic cell death. In cancers with mutated *TP53*, the efficiency of drugs inducing inhibition of MDM2 activity should be explored. Indeed, the activity of many MDM2 inhibitors, even though not yet approved by the regulatory authorities, is under investigation in clinical (130, 178). In this context, a new very promising approach to neutralize MDM2 has been recently proposed by using a proteolysis targeting Chimera for MDM2 targeting and degradation on p53-inactivated triple-negative breast cancer cells, which was highly effective in the induction of apoptotic cell death (179).

**TABLE 2:** Effect of RiBi inhibitors on cancer cells depending on ribosome biogenesis and RP mutations/deletions.

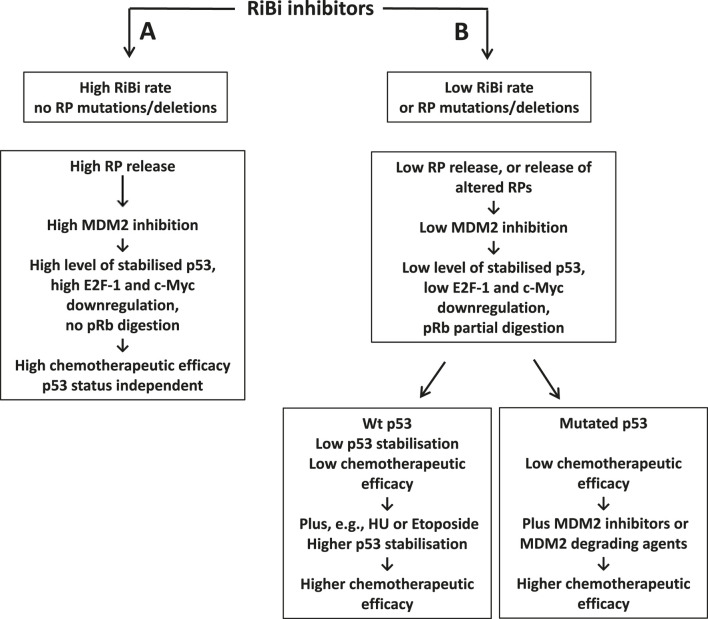

A, The inhibition of ribosome biogenesis in cells with a high level of RiBi rate causes a marked inhibition of oncogenic activities of MDM2 leading to a strong p53 stabilization, a marked E2F-1 and c-Myc downregulation and a pRb preservation. These effects are responsible for a high chemotherapeutic efficacy of RiBi inhibitors on cancers with both WT and mutated p53.

B, The inhibition of ribosome biogenesis in cells with a low RiBi rate or harboring mutated/deleted ribosomal proteins involved in the control of MDM2 is responsible for a low level p53 stabilization, a low E2F-1 and c-Myc downregulation and a pRb partial preservation, thus being responsible of a low chemotherapeutic efficacy. In order to increase the chemotherapeutic efficacy on cancers with a low RiBi rate, and with a WT p53, drugs stabilizing p53 through a different way than that of RP-MDM2-p53 pathway (such as Hydroxyurea or Etoposide) should be added to RiBi inhibitors. In the case of cancers with mutated p53, the possibility of adding MDM2 inhibitors or MDM2-degrading agents might be considered in order to increase the chemotherapeutic efficacy.

In conclusion, the evaluation of the ribosome biogenesis rate and the analysis of the presence of mutations/deletions of RPs in cancer tissues should be of great utility in order to establish adequate anti-cancer chemotherapeutic protocols (see also Table 2).
